# Does Massage Gun or Foam Roller Use During a Warm-Up Improve Performance in Trained Athletes?

**DOI:** 10.3390/sports13090282

**Published:** 2025-08-22

**Authors:** Lachlan Ormeno, Matthew Driller

**Affiliations:** Sport, Performance, and Nutrition Research Group, School of Allied Health, Human Services, and Sport, La Trobe University, Melbourne 3086, Australia

**Keywords:** vibration therapy, percussive massage, range of motion, athletic performance

## Abstract

Self-myofascial release tools like foam rollers and massage guns are being integrated into athlete warm-ups with increasing frequency, but evidence on their acute effects is limited. Sixteen healthy, trained athletes (23.2 ± 1.3 years; four female) completed three warm-up conditions in a randomised, crossover design separated by >48 h: dynamic warm-up plus foam rolling (FOAM), dynamic warm-up plus massage gun (GUN), and dynamic warm-up alone (CON). After each intervention, participants completed a countermovement jump (CMJ; height and reactive strength index [RSImod]), a 10/5 repeated jump test (RJT), a 20 m sprint, and a knee-to-wall ankle mobility test. Perceived soreness and fatigue were recorded. Linear mixed models and Cohen’s *d* were used to assess between-condition differences. Relative to CON, FOAM and GUN were associated with reduced CMJ height (*d* = −0.29 to −0.36) and RSImod (*d* = −0.40 to −0.52; *p*’s < 0.05). GUN was associated with significantly impaired sprint time (*d* = 0.34). There were modest improvements in ankle mobility (left side) following FOAM (*d* = 0.23, *p* < 0.05) and lower levels of muscle soreness compared to CON (*p* < 0.05). Despite some improvements in ankle mobility and muscle soreness with foam rolling, both foam rolling and massage gun use may acutely impair aspects of physical performance compared to a dynamic warm-up alone.

## 1. Introduction

Warming up prior to physical activity is a well-established practice aimed at enhancing performance and reducing injury risk through a combination of physiological and neuromuscular mechanisms [1]. Traditional warm-up protocols often incorporate aerobic activity, dynamic stretching, and mobility exercises to increase core temperature, muscle elasticity, and neural activation [2]. More recently, self-myofascial release (SMR) techniques, such as foam rolling, have been integrated into warm-up and recovery routines with increasing frequency due to their potential to improve range of motion, reduce muscle stiffness, and mitigate delayed-onset muscle soreness [3,4]. Similarly to foam rolling, the use of massage guns, or percussive massage therapy, has been proposed as a technique to elicit similar proposed benefits [5].

The use of handheld massage guns has gained popularity among athletes as a tool for recovery [6,7]. These devices apply high-frequency vibrations and percussive forces at variable amplitudes, with proposed benefits including increased blood flow, reduced muscle stiffness, improved tissue hydration, and enhanced joint mobility [8,9]. A study by Leabeater et al. (2022) found that 15% to 25% of competitive triathletes incorporate massage guns into their weekly routines, primarily for post-exercise recovery and muscle soreness management [7]. Menek & Menek (2024) expanded on this by examining the acute effects of percussive therapy, finding that while a brief application improved hip flexion range of motion (ROM) and reduced perceived muscle stiffness, it did not enhance immediate muscle strength or power output [6]. Ferreira et al. (2023) conducted a systematic review highlighting that massage guns can effectively increase local blood flow, improve soft tissue compliance, and reduce perceived soreness, though their influence on delayed-onset muscle soreness (DOMS) and performance outcomes remain mixed when compared to other recovery tools [8]. In contrast, a systematic review of 13 studies by Sams et al. (2023) recommended that percussive therapy delivered by massage guns can help improve acute muscle strength, explosive muscle strength, and flexibility [10]. More recently, Leabeater et al. (2024) evaluated their use as a recovery tool, reporting modest improvements in joint mobility, but no significant enhancement in performance measures such as sprint speed or jump height when compared to traditional dynamic warm-ups [9]. Despite their widespread use, much of the current literature on percussive therapy has focused on post-exercise recovery, with limited evidence supporting its effectiveness as a warm-up modality.

Foam rolling, on the other hand, has been more extensively investigated in both recovery and warm-up contexts, allowing for a better understanding of its effects on performance variables such as power, flexibility, and muscular efficiency [6,11]. McCary et al. (2015) found that foam rolling was associated with enhanced joint ROM, particularly in the lower limbs, providing a key benefit to athletes, while also improving overall muscle efficiency during subsequent activities. The study indicated that when used as a warm-up tool it helps improve flexibility without compromising power or explosive strength [11].

Mechanistically, both interventions appear to acutely improve soft tissue elasticity via the vibrational pressure provided by the massage gun or mechanical pressure from the foam roller on the fascia and underlying musculature [12]. This may lead to enhanced joint range of motion (ROM) without compromising muscle strength or power output, in contrast to static stretching [13]. Increased ROM may reduce injury risk by enabling greater flexibility and improved movement mechanics during activity [14]. Another proposed mechanism is enhanced blood flow and local muscle temperature, and these are attributed to the vibrational or mechanical pressure applied during massage gun use or foam rolling [4]. These mechanisms elevate tissue temperature and promote perfusion, which facilitates faster circulatory system reactions and improved muscle viscosity, to collectively prepare the muscles for movement [15]. From a neurological perspective, foam rolling and percussive massage may modulate pain perception and reduce perceived muscle soreness prior to exercise [16,17]. These effects could contribute to improved ROM and demonstrate potential higher performance outputs by reducing discomfort that might otherwise limit effort. Together, these mechanical, circulatory, and neural adaptations suggest that massage guns and foam rolling, when used appropriately in a warm-up, may acutely improve mobility, enhance muscle performance, and lower injury risk [4,15,16,17].

Although several recent investigations have directly compared foam rolling and vibration-based or percussive modalities, none have examined a standard, non-vibration roller versus a handheld massage gun device as an acute warm-up strategy. For example, Wang et al. (2022) showed that vibration foam rolling and local mechanical vibration applied during warm-up produced small gains in tennis-specific performance [18]. In professional soccer players, Padrón-Cabo et al. (2024) found that vibration and non-vibration foam rolling elicited similar increases in muscle perfusion [19], and Secer and Kaya (2025) reported comparable improvements in fitness parameters after six weeks of vibration versus non-vibration rolling [19]. Likewise, Nakamura et al. (2022) and Reiner et al. (2021) observed near-identical effects on plantar-flexor and quadriceps function, respectively, when foam rolling was augmented with vibration [20,21]. Importantly, all of these studies either used vibration-equipped rollers or assessed recovery and longer-term adaptations, not the immediate, self-administered use of a non-vibration foam roller and percussive massage gun as part of a pre-exercise warm-up. By directly comparing these two tools within the warm-up sequence, the present study fills a clear gap in our understanding of how self-myofascial modalities influence acute performance outcomes.

Therefore, the primary objective of this study was to investigate the acute effects of massage guns and foam rollers during a warm-up on physical performance metrics in trained athletes. Specifically, this research aimed to examine how each modality influences key performance outcomes such as strength, power, speed and mobility when used as part of a pre-activity routine. This represents a notable gap in the literature, particularly given the common substitution of these modalities in applied settings. Understanding these effects will provide athletes, coaches, and practitioners with evidence-based guidance on the efficacy of these tools within warm-up protocols.

## 2. Materials and Methods

### 2.1. Participants

A total of 16 healthy active young adults (age 23.19 ± 1.30 y; 4 females; 12 males) volunteered to participate in this study. All participants were classified as Tier 2, trained athletes (regularly training ~3 times per week for a specific sport) [22]. Participants were all team-sport athletes (Australian rules football: n = 6; basketball: n = 5; netball: n = 4; soccer: n = 1), had at least 3 years of strength-training experience, and had trained in the gym a minimum of 2 times per week over the last 6 months prior to taking part in the study. All participants were injury-free in their lower limbs (hip, knee, or ankle) and free of any other injuries that may have affected their ability to perform the physical tests or warm-up protocols. Inclusion criteria required participants to be 18–30 years of age and both male and female athletes were recruited. A simulation-based power analysis was conducted in R (v4.3.1) using the simr package (v1.0.6) for our planned linear mixed-effects model (Outcome ~ Condition + (1 | Participant)). We anchored our estimate on the moderate CMJ effect reported by Drinkwater et al. (2019), who observed Hedges’ g = 0.54 for post-foam rolling jump improvements in trained males (equivalent to f ≈ 0.27) [23]. Assuming residual variance = 1 and random-intercept variance = 0.2, we generated 1000 simulated datasets for N = 16 subjects measured under three conditions. The fixed effect of Condition was detected at α = 0.05 in 80.3% of simulations, demonstrating that 16 participants provide ≥ 80% power to detect our target effect within the linear mixed model (LMM) framework. All participants provided written informed consent, and the study was approved by the Institutions Human Research Ethics Committee (#HEC25042).

### 2.2. Design

**Figure 1:**
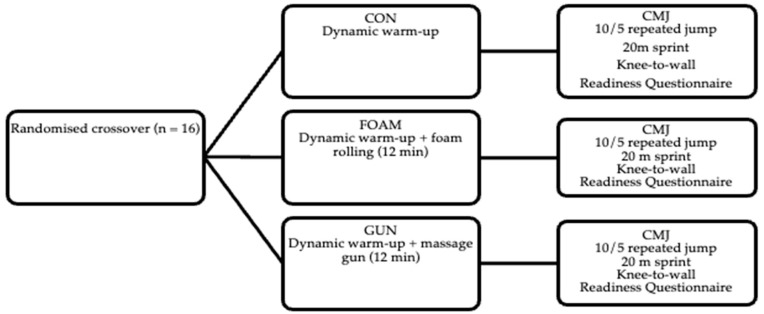
Experimental design including the three conditions: control (CON); foam-rolling (FOAM); and massage gun (GUN). CMJ = countermovement jump. All physical and perceptual measures were completed only once, following each warm-up protocol.

The current study adopted a randomised, counterbalanced crossover design, with three experimental trials (massage gun [GUN] and foam rolling [FOAM]) and a control trial (dynamic warm-up only [CON]). The order of trials was randomised and counterbalanced using a 3 × 3 Latin-square design: three sequences (A→B→C; B→C→A; C→A→B) were generated and assigned in rotating fashion so that each protocol appeared exactly once in the first, second, and third position across the cohort. We opted for this experimental design because this approach maximises statistical power by using within-subject comparisons and reduces the error variance associated with inter-individual differences. All participants completed a standardised dynamic warm-up in each condition before the GUN and FOAM participants completed their standardised protocol. Participants completed a perceptual questionnaire immediately following the warm-up, and physical tests 3 min after warm-up protocols (Figure 1). Participants completed the entire study in three separate testing sessions, each separated by a minimum of 2 days, and a maximum of 5 days. Participants were asked to refrain from exercise within a 24 h period prior to each session to limit any influence on physical testing. Participants were also asked to refrain from ingesting caffeine or other pre-workout supplements in the 3 h prior to testing. All participants had prior experience with both foam rollers and massage guns before taking part in the study.

### 2.3. Procedures

A dynamic warm-up was performed by all participants at the beginning of each testing session to standardise every session [24]. This dynamic warm-up was created following the RAMP (Raise, Activate, Mobilise, Potentiate) system, as outlined by Jeffreys [25]. This consisted of dynamic exercises progressing from low-intensity, generic movements to high-intensity movements and more specific movements related to the testing protocol (Table 1).

### 2.4. Massage Gun

**Figure 2:**
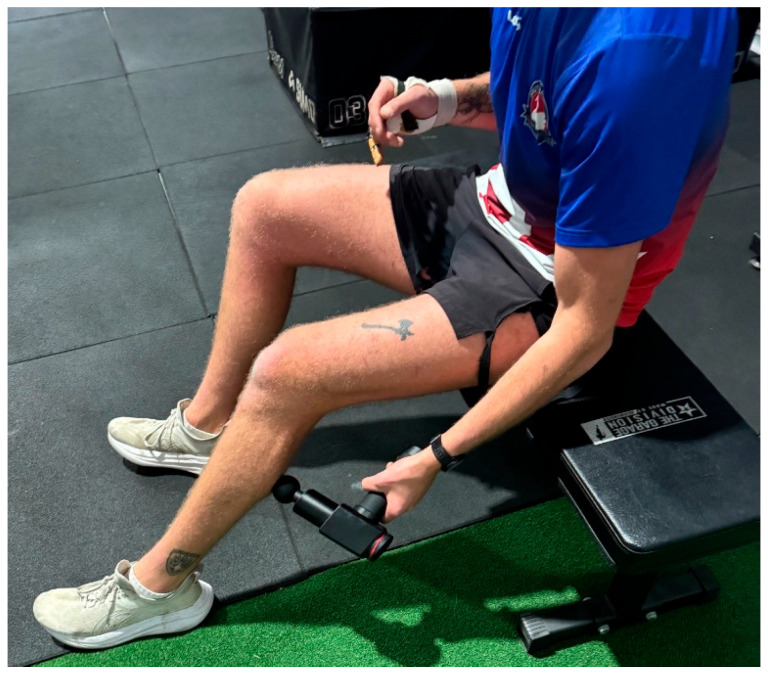
Participant completing massage gun application on left lower limb.

The GUN intervention involved a 12 min (6 min per leg) treatment of the quadriceps, hamstrings, and calf muscles using a massage gun device (Hydragun, Farmingdale, NY, USA) with a soft attachment (a ball-shaped head). The massage gun was used at a speed of 53 Hz, or approximately 3200 rpm [26]. Participants were instructed to apply the massage gun to the quadricep, then the hamstring, and lastly the calf, while seated (Figure 2). A timer was displayed for two minutes for each landmark on a chosen leg before the participants switched to the opposite leg to complete the same protocol. As per the manufacturer’s instructions, participants were directed to apply moderate pressure and to “glide” the massage gun along the muscle belly continuously at the selected amplitude, covering the muscles at a speed of approximately 2 cm per second [27]. This method of prescribing time for each lower limb has been shown to significantly increase range of motion and significantly decrease passive stiffness [28]. Researchers supervised the intervention to ensure correct placement and consistency between participants.

### 2.5. Foam Rolling

**Figure 3:**
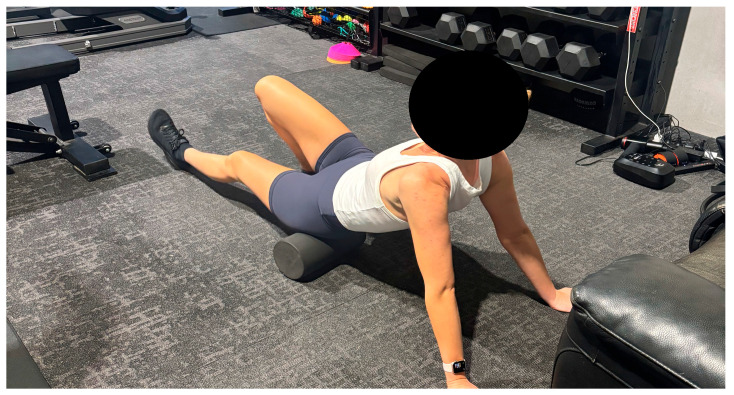
Participant completing foam rolling on their left hamstring.

The FOAM intervention involved a 12 min (6 min per leg) treatment of the quadriceps, hamstrings, and calf muscles using a foam roller (Figure 3). A high-density EVA roller (Crane, Ipswich, UK; 30 cm length × 15 cm diameter) was used. Participants were instructed to apply the foam roller to the quadricep, then the hamstring, and lastly the calf while on the ground, maintaining a pressure of ~25% bodyweight, as suggested previously [29]. A timer was displayed for two minutes for each landmark on a chosen leg before switching to the opposite leg to complete the same intervention. This method of prescribing time to each landmark has been shown to significantly increase range of motion and muscle strength [30]. Researchers supervised the intervention to ensure correct placement and consistency was maintained between participants.

### 2.6. Athlete Readiness Questionnaire

Using a modified McLeans questionnaire (comprising only two of the five items), participants rated their fatigue and muscle soreness on a scale of 1 to 5 [31], where for fatigue, 1 = always tired, while 5 = very fresh, and for muscle soreness, 1 = very sore and 5 = feeling great. Ratings were obtained immediately after participants completed their warm-up protocols (see Figure 1 above).

### 2.7. Countermovement Jump Test

The countermovement jump test (CMJ) is performed as a measure of lower-body power and neuromuscular performance. The CMJ is a widely validated and reliable assessment in athletic populations, demonstrating strong inter- and intra-reliability, with coefficients of variation that are typically less than 5% and intraclass correlation coefficients (ICCs) exceeding 0.90 when performed under standardised conditions [32,33]. Standardised protocols for the CMJ involve participants starting in an upright position with hands on their hips to eliminate arm swing, followed by a rapid downward movement to a self-selected depth and immediate upward jump for maximal height [34]. Participants performed three maximal jumps. The jumps are conducted using ForceDecks Lite (VALD Performance, Brisbane, Australia), sampling at 1000 Hz. The best trial of all jumps will be used for analysis. Participants’ jump height and reactive strength index modified (RSImod) were taken from their best trial [32]. RSImod was used in addition to jump height to determine the participants’ ability to quickly produce force relative to the time they took to initiate a jump. This procedure has been shown to produce a highly reliable measure of explosive lower-body power [34].

### 2.8. The 10/5 Repeated Jump Test

The 10/5 repeated jump test (RJT) is performed as a measure of reactive strength and stretch-shortening cycle efficiency post warm-up. Previous studies demonstrated excellent reliability and validity in athletic populations, with ICCs ranging from 0.86 to 0.98 [35]. Participants executed 10 consecutive maximal vertical jumps while minimising ground contact time [36]. Metrics including RSImod, flight time, and contact jump were recorded from the best five jumps and used for analysis.

### 2.9. The 20 m Sprint Test

A 20 m sprint was performed as a measure of speed using SmartSpeed (VALD Performance, Brisbane, Australia) equipment [37,38]. The 20 m sprint tests the participants ability to complete the 20 m in the fastest amount of time possible (measured in seconds) [37]. This test is widely recognised for its high test–retest reliability in assessing short-distance sprint performance [39].

### 2.10. Knee-to-Wall Lunge Test (Ankle Mobility)

The knee-to-wall test (KTW) was performed as a reliable measure of ankle mobility/range of motion (ROM) in a weight-bearing position, which is essential for running and jumping biomechanics [40]. The KTW has demonstrated excellent reliability, with an ICC between 0.88 and 0.99 in healthy adults [41]. Participants stood with one foot centred on a tape measure secured to the floor and aligned perpendicular to a wall. Keeping both the heel and toe on the tape’s midline, participants gradually stepped the toe farther from the wall and performed repeated lunges until they could no longer touch their knee to the wall without lifting their heel. The maximal distance from the tip of the big toe to the wall was then recorded (in centimetres).

### 2.11. Statistical Analysis

Analyses were conducted using IBM SPSS Statistics (version 29). A one-way repeated measures design was used to assess the effects of three conditions (CON, GUN, and FOAM) on a range of performance and perceptual variables. Linear mixed models (LMMs) were employed to account for the repeated-measures nature of the data, with Condition modelled as a fixed effect and Participant as a random effect. The models were fit using Restricted Maximum Likelihood (REML) estimation. This model was selected for its parsimony and appropriateness for repeated-measures data, as it accounts for inter-individual variability while maintaining statistical power. Model comparisons were conducted to justify the random-intercept model over a random-intercept-and-slope model, using Akaike Information Criterion (AIC), Bayesian Information Criterion (BIC), and likelihood-ratio tests, favouring the simpler random-intercept model. Pairwise comparisons between conditions were conducted using Bonferroni-adjusted estimated marginal means. Normality of residuals was assessed using the Shapiro–Wilk test and visual inspection of residual plots. Residuals were approximately normal for most variables, with the exception of 10/5 Contact Time (*p* = 0.005). Homoscedasticity was confirmed via residuals-vs.-fitted plots, showing no systematic patterns. Given the robustness of linear mixed models with regard to moderate deviations from normality, all analyses were retained as planned. Cohen’s *d* was calculated for significant pairwise comparisons to quantify the magnitude of effects. Effect size thresholds were interpreted as *small* (*d* = 0.2), *medium* (*d* = 0.5), and *large* (*d* = 0.8).

## 3. Results

**Figure 4:**
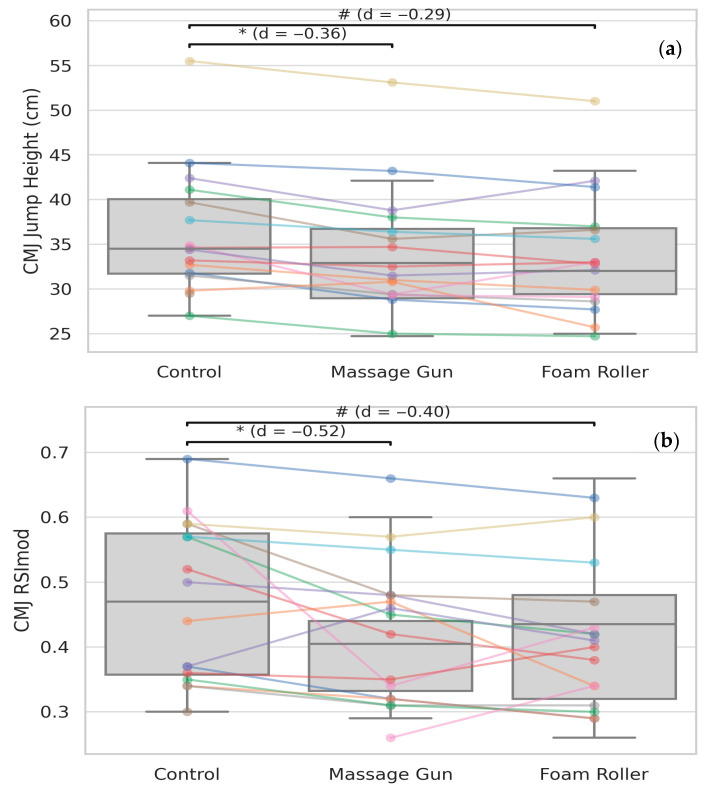
(**a**) CMJ height (cm) and (**b**) CMJ RSImod across the three conditions. Boxplots with individual participant data points and connecting lines are shown. Significant differences are annotated as follows: * indicates a significant difference between massage gun and control (*p* < 0.05); # indicates a significant difference between foam roller and control (*p* < 0.05); Cohen’s *d* values indicate the magnitude of the observed effect.

Linear mixed-effects models were employed to analyse the data, revealing the main statistically significant effects of the experimental conditions on multiple outcome variables. Specifically, for CMJ height, both the GUN condition (*p* < 0.01, *d* = −0.36) and the FOAM condition (*p* < 0.01, *d* = −0.29) demonstrated significantly reduced jump heights compared to the control condition (CON). These results are detailed in Table 2 and visually represented in Figure 4. Similarly, for the CMJ RSImod, both the GUN condition (*p* < 0.01, *d* = −0.52) and the FOAM condition (*p* < 0.01, *d* = −0.40) exhibited significantly lower values relative to the CON condition, as presented in Table 2 and Figure 4. These findings indicate that both GUN and FOAM interventions led to measurable reductions in performance outcomes compared to the baseline control, with *small*-to-*moderate* effect sizes observed across the variables.

**Figure 5:**
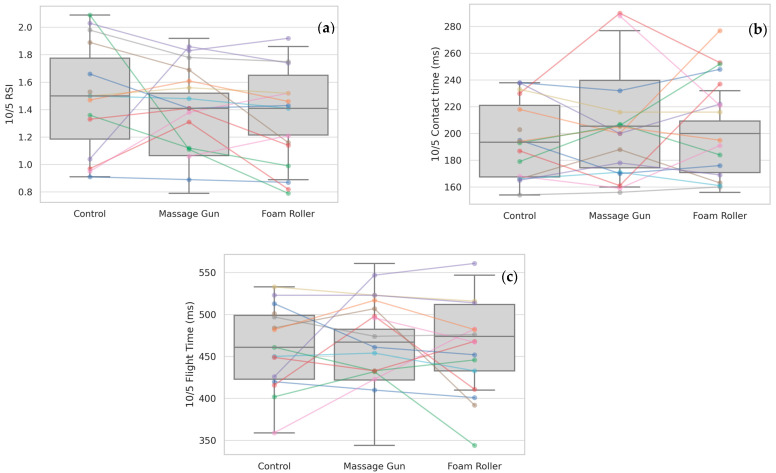
(**a**) 10/5 RSI; (**b**)10/5 contact time; and (**c**) 10/5 flight time across the three conditions. Boxplots with individual participant data points and connecting lines are shown.

No significant differences were observed for 10/5 RSI (*p* = 0.276), 10/5 Contact Time (ms) (*p* = 0.477), or 10/5 Flight Time (ms) (*p* = 0.348) (Table 2, Figure 5).

**Figure 6:**
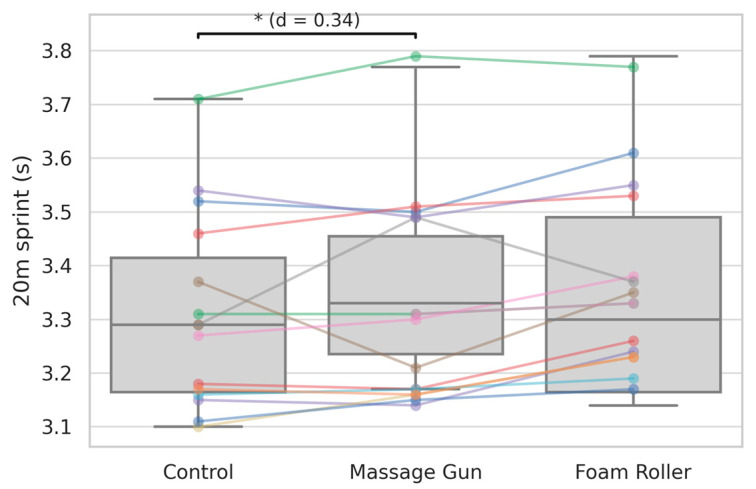
A 20 m sprint across the three conditions. Boxplots with individual participant data points and connecting lines are shown. Significant differences are annotated as follows: * indicates a significant difference between massage gun and control (*p* < 0.05); Cohen’s *d* values indicate the magnitude of the observed effect.

For 20 m sprint time, a significant main effect was observed (*p* < 0.01). GUN resulted in significantly slower sprint times compared to CON (*p* < 0.01, *d* = 0.34), while FOAM did not differ significantly from CON (Table 2, Figure 6).

For ankle mobility (Left), FOAM (*p* = 0.03, *d* = 0.31) showed significantly greater mobility compared to CON (Table 2). There were no significant interactions for right ankle mobility.

For muscle soreness, FOAM resulted in significantly lower muscle soreness scores compared to CON (*p* < 0.01, *d* = 0.87), with no other significant differences between trials. There were no significant differences between trials for self-reported fatigue (Table 3).

## 4. Discussion

A 12 min intervention of a commercially available massage gun or foam roller immediately after a dynamic warm-up led to significant decreases in CMJ height and CMJ RSImod compared to the control trial. Similarly, the massage gun trial resulted in significantly slower 20 m sprint times relative to control, while foam rolling had no significant effect on sprint performance. Left-side ankle mobility significantly increased joint ROM following foam rolling compared to the control and was associated with significantly less muscle soreness. Collectively, these findings suggest that massage gun and foam rolling may impair some measures of physical performance when compared to a dynamic warm-up alone, despite modest improvements in ROM and muscle soreness.

The main outcome was that massage guns and foam rollers appear to limit physical performance after a warm-up compared to a CON condition. The observed reductions in CMJ performance and sprint speed following GUN and FOAM interventions are broadly consistent, with some previous reports showing performance decrements following passive or mechanical interventions [4,5,8,42]. Some studies, however, reported improved or unchanged performance following foam rolling or percussive interventions [12,43]. The present finding reinforces the notion that, despite potential benefits for mobility, massage gun and foam rolling may not be optimal immediately prior to activities requiring maximal power output.

The finding that left ankle mobility improved following FOAM use aligns well with the existing literature. Numerous studies have demonstrated that modalities like foam rolling can acutely enhance joint range of motion through mechanisms such as myofascial relaxation, changes in tissue viscoelasticity, and pain modulation [13,44]. However, increased ankle mobility over time, particularly dorsiflexion, has been linked to an increase in injury prevention as it improves jumping and sprinting biomechanics and reduces compensatory movements in knees and hips, whilst also providing more joint stability for lateral sprains [45,46,47,48]. Furthermore, a study investigating the immediate effects of ankle mobilisation found a significant improvement in ankle ROM and reductions in knee pain immediately after the intervention, indicating that acutely increasing ankle ROM does reduce compensatory movement and improve joint stability [49]. These mobility gains may be desirable in contexts where range of motion is limiting performance or where flexibility is the primary goal of the warm-up. However, given that these changes were minimal (<1 cm), and only on the left ankle following FOAM, it is important to consider these in context.

Interestingly, neither condition affected 10/5 RSI or contact/flight time. These findings suggest that measures of reactive strength and rapid stretch-shortening cycle performance may be less sensitive to the acute effects of foam rolling and massage gun use, or that the applied protocols were insufficient to induce measurable changes in these outcomes. Previous findings are inconsistent, with other studies also showing non-significant decreases in jump metrics [4,5]. However, percussive therapies like massage guns have been shown to significantly improve jumping metrics compared to foam rolling [5].

Muscle soreness scores were significantly lower in the FOAM condition compared to CON; however, no significant differences in soreness appeared when using the massage gun. The foam rolling results are consistent with previous studies that show that it can alleviate muscle soreness whilst also significantly reducing delayed-onset muscle soreness (DOMS) [4,50]. Percussive massage treatment has shown inconsistent results for muscle soreness, with some studies indicating increased muscle soreness after use [9], whilst others show decreases in muscle soreness [51,52].

Interestingly, there were no significant differences for muscle fatigue in FOAM and GUN conditions compared to CON. This is not consistent with previous studies that show muscle fatigue improves pre–post use of such interventions [4,5,8,50,51,53]. However, previous study interventions have assessed these modalities as post-exercise recovery strategies, rather than strategies to be used during a warm-up.

This study presents several methodological and practical strengths that enhance the validity and relevance of its findings. The use of a randomised, counterbalanced crossover design minimises potential order effects and allows each participant to act as their own control, thereby reducing inter-individual variability. Secondly, the inclusion of multiple performance measures, including physical tests (CMJ, 10/5 RJ, 20 m sprint, and knee to wall), paired with a perceptual measure (muscle soreness and fatigue), provides a comprehensive evaluation of both physiological and subjective responses to the modalities. Third, all data pertaining to outcome measures were collected using validated and reliable tools, such as ForceDecks Lite and SmartSpeed timing gates, further supporting data accuracy and reproducibility [54,55]. Additionally, the sample consisted of trained athletes from various sporting backgrounds, which strengthens the external validity and applicability of the findings across different athletic contexts. Lastly, standardised protocols for dynamic warm-up and intervention timing ensured consistency across all sessions.

Several limitations should be considered when interpreting this study’s results. First, there is no consensus on the recommended vibration speed, time frame, or amplitude of massage guns, making it difficult to generalise our findings. Future research on both foam rolling and massage gun use should adhere to the protocols and recommendations proposed by Ferreira et al. [8,56]. Second, the study focused on short-term responses only, and did not assess longer-term responses or the potential cumulative effects of repeated use. Future research should also consider performing these interventions prior to the dynamic warm-up. Thirdly, the study sample comprised trained individuals, which may limit the generalisability to other populations (e.g., elite athletes and novices). Lastly, we did not collect any follow-up data on muscle soreness in the days after the GUN or FOAM interventions. Although sessions were separated by ≥48 h to allow recovery, residual discomfort could still have influenced performance in a subsequent condition. Future studies should include systematic post-session soreness assessments (e.g., via visual analogue scales) and consider extending the washout period (e.g., >72 h) to fully eliminate any carryover effects. Carryover effects should also be measured using baseline fatigue and soreness questionnaires.

Future research should explore the effects of regular daily massage gun and foam roller use on performance and mobility. It would also be valuable to assess dose–response relationships, examining how the duration, intensity, and timing of these interventions modulate their effects. Finally, further work should investigate their impact in different populations and across various types of athletic performance.

This research provides the first comparative analysis of massage gun and foam roller use during warm-ups in a controlled experimental setting. The findings support the notion that using massage guns and foam rollers acutely prior to explosive exercise may impair aspects of physical performance. While evidence suggests that foam rolling may result in modest increases in ankle ROM and lower muscle soreness levels, their use immediately prior to explosive performance may compromise outcomes such as jump height and sprint speed.

## Figures and Tables

**Table 1:** Dynamic warm-up protocol.

Exercise	Reps
High knees	20
Butt kicks	20
Star jumps	20
Floor glute bridges	8
Deep bodyweight squat	10
Lunge and back w/overhead reach	8 each side
CMJs 50–100%	5
Pogos	20

**Table 2:** Means ± SD for physical performance tests following each condition (CON, GUN, and FOAM), with significant pairwise comparisons indicated.

Variable	Con Mean ± SD	Gun Mean ± SD	Foam Mean ± SD	*p*-Value (LMM)
CMJ Jump Height (cm)	36.24 ± 7.08	33.77 ± 6.83 *	34.22 ± 6.82 #	<0.01
CMJ RSImod	0.47 ± 0.12	0.41 ± 0.10 *	0.42 ± 0.11 #	<0.01
10/5 RSI	1.48 ± 0.40	1.32 ± 0.35	1.43 ± 0.30	0.276
10/5 Contact time (ms)	195.44 ± 28.61	207.81 ± 38.04	201.69 ± 40.58	0.447
10/5 Flight Time (ms)	461.07 ± 49.77	456.40 ± 55.03	475.40 ± 43.50	0.348
20 m sprint (s)	3.31 ± 0.18	3.37 ± 0.17 * ^	3.32 ± 0.19 ^	<0.01
Ankle Mobility L (cm)	12.30 ± 2.53	12.83 ± 1.99	13.00 ± 1.91 #	0.03
Ankle Mobility R (cm)	12.81 ± 2.35	13.16 ± 1.73	13.31 ± 1.86	0.11

Note: * indicates a significant difference between GUN and CON (*p* < 0.05); # indicates a significant difference between FOAM and CON (*p* < 0.05); ^ indicates a significant difference between GUN and FOAM (*p* < 0.05). Overall *p*-values are based on the linear mixed model (LMM).

**Table 3:** Means ± SD for perceptual measures following each condition (CON, GUN, and FOAM), with significant pairwise comparisons indicated. A higher score for both fatigue and muscle soreness indicates that participants are feeling very fresh (fatigue) and feeling great (muscle soreness). LMM—linear mixed model.

Variable	Con Mean ± SD	Gun Mean ± SD	Foam Mean ± SD	*p*-Value (LMM)
Muscle Soreness (/5)	3.75 ± 0.45	4.12 ± 0.89	4.31 ± 0.79 #	<0.01
Fatigue (/5)	4.13 ± 0.81	4.06 ± 0.57	4.13 ± 0.89	0.960

Note: # indicates a significant difference between FOAM and CON (*p* < 0.05). Overall *p*-values are based on the linear mixed model.

## Data Availability

The data that support the findings of this study are available on reasonable request to the corresponding author. The data are not publicly available due to privacy and ethical restrictions.
